# Deep Convolutional Neural Network Used in Single Sample per Person Face Recognition

**DOI:** 10.1155/2018/3803627

**Published:** 2018-08-23

**Authors:** Junying Zeng, Xiaoxiao Zhao, Junying Gan, Chaoyun Mai, Yikui Zhai, Fan Wang

**Affiliations:** School of Information Engineering, Wuyi University, Jiangmen 529020, China

## Abstract

Face recognition (FR) with single sample per person (SSPP) is a challenge in computer vision. Since there is only one sample to be trained, it makes facial variation such as pose, illumination, and disguise difficult to be predicted. To overcome this problem, this paper proposes a scheme combined traditional and deep learning (TDL) method to process the task. First, it proposes an expanding sample method based on traditional approach. Compared with other expanding sample methods, the method can be used easily and conveniently. Besides, it can generate samples such as disguise, expression, and mixed variation. Second, it uses transfer learning and introduces a well-trained deep convolutional neural network (DCNN) model and then selects some expanding samples to fine-tune the DCNN model. Third, the fine-tuned model is used to implement experiment. Experimental results on AR face database, Extend Yale B face database, FERET face database, and LFW database demonstrate that TDL achieves the state-of-the-art performance in SSPP FR.

## 1. Introduction

As artificial intelligence (AI) becomes more and more popular, computer vision (CV) also has been proved to be a very hot topic in academic such as face recognition [1], facial expression recognition [2], and object recognition [3]. It is well known that the basic and important foundation in CV is that there are an amount of training samples. But in actual scenarios such as immigration management, fugitive tracing, and video surveillance, there may be only one sample, which leads to single sample per person (SSPP) problem such as gait recognition [4], face recognition (FR) [5, 6], and low-resolution face recognition [7] in CV. However, as the widely use of second-generation ID card which is convenient to be collected, SSPP FR becomes one of the most popular topics no matter in academic or in industry.

Beymer and Poggio [8] proposed one example view problem in 1996. In [8], it was researched that how to perform face recognition (FR) using one example view. Firstly, it exploited prior knowledge to generate multiple virtual views. Then, the example view and these multiple virtual views were used as example views in a view-based, pose-invariant face recognizer. Later, SSPP FR became a popular research topic at the beginning of the 21st century.

Recently, many methods have been proposed. Generally speaking, these methods can be summarized in five basic methods: direct method, generic learning method, patch-based method, expanding sample method, and deep learning (DL) method. Direct method does experiment based on the SSPP directly by using an algorithm. Generic learning method is the way that using an auxiliary dataset to build a generic dataset from which some variation information can be learned by single sample. Patch-based method partitions single sample into several patches first, then extracts features on these patches, respectively, and does classification finally. The expanding sample method is with some special means such as perturbation-based method [9, 10], photometric transforms, and geometric distortion [11] to increase sample so that abundant training samples can be used to process this task. The DL method uses the DL model to perform the research.

**Figure 1:**
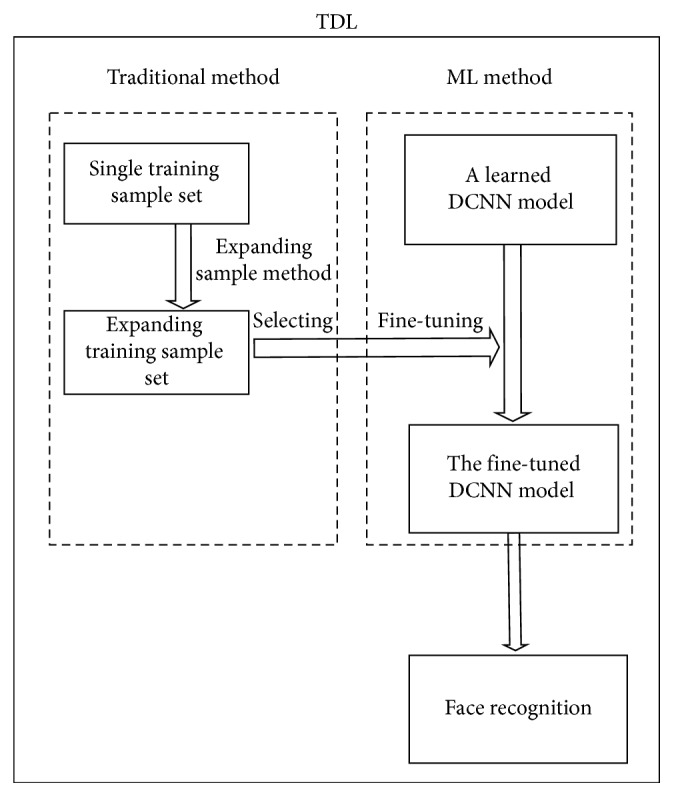
The framework of the proposed method.

Attracted by the good performance of DCNN, inspired by [12] and driven by AI, in this paper, a scheme combined traditional and DL (TDL) method is proposed. The framework of TDL is illuminated in Figure 1. First, an expanding sample method is proposed to increase the sample to overcome the shortage of sample in SSPP FR. Second, a learned DCNN model is brought in, and then some expanding samples are selected to fine-tune the model. Finally, the fine-tuned model is used to perform experiment.

This is an extended version of our conference papers [13, 14]. The contributions of this paper are shown as follows:

- We propose a novel expanding sample method. Compared with other expanding sample methods, it is more easier and convenient to be used. Besides, the expanding sample method can generate expression, disguise, and mixed variation which other expanding sample methods cannot achieve.
- We use DCNN to perform SSPP FR. Here, we propose bringing transfer learning into SSPP FR to avoid the requirement of training DCNN that needs abundant samples.
- We propose TDL, that is, combined traditional and DL method to do this task. Firstly, we select images from expanding samples to fine-tune the DCNN model. Then, the fine-tuned DCNN model is used to implement experiment.
- We construct an intraclass variation set which can be used anywhere to expand facial sample.

The remaining parts of the paper are structured as follows.  Session 2 introduces related works.  Session 3 presents the expanding sample method.  Session 4 presents the deep learning method.  Session 5 implements experiments.  Session 6 concludes the paper and indicates the future work.

## 2. Related Works

In recent years, many scholars in the world devoted themselves to SSPP FR, and some good performances were obtained. Deng et al. [15] proposed extended sparse representation-based classifier (ESRC) method to classify query sample and gallery sample. With the help of an auxiliary training set, it used variations of the auxiliary training set to represent those that lack variations of the gallery set. Lu et al. [16] proposed a novel discriminative multimanifold analysis (DMMA) method. It obtained patches of training sample by segmenting image, and then these patches were used to learn discriminative features. Mohammadzade and Hatzinakos [17] learned expression invariant subspace to keep expression invariant. It pointed out that the same expression has the same expression subspace, and it can generate a new image by projecting an expression image to expression subspace. Yang et al. [18] proposed sparse variation dictionary learning (SVDL) method. It connected generic set and gallery set adaptively by jointly learning a projection, rebuilding a sparse dictionary including adequate variations, and performing SSPP FR by projecting variation dictionary to gallery set space. Li et al. [19] developed linear discriminant analysis (LDA) to process the SSPP FR problem and produced extrauseful training samples in low-dimension subspace by using random projection. Zhu et al. [6] proposed a framework based on local generic representation to solve the SSPP FR problem. It used the same way as ESRC to build intraclass variation dictionary and proportioned the face image into several patches to extract local information. Liu et al. [20] proposed a fast FR method based on DMMA. First, it clustered two groups of persons using a rectified K-means method. Second, it partitioned the face image into several nonoverlap patches, and then DMMA was applied on these patches. Third, fast DMMA was obtained by repeating the former two steps. Liu et al. [21] solved the SSPP FR problem by using sparse representation-based classifier (SRC) and local structure. It relieved the trouble that had high-dimension data and few samples. Mokhayeri et al. [22] expanded the training set by using an auxiliary set. Gao et al. [23] presented a regularized patch-based representation method. A collection of patches are used to represent each image; meanwhile, under the gallery image patches and intraclass variance dictionaries, their sparse representations are sought. Song et al. [5] proposed a triple local feature-based collaborative representation method to make full use of the training sample. First, it extracted different types of Gabor features including different scales and different directions. Second, it partitioned each Gabor feature into several local patches to obtain triple local features including local scale, local direction, and local space. Third, it did local collaborative representation and classification based on these triple local features. Zhang and Peng [24] used deep autoencoder to generalise intraclass variations, and then these intraclass variations were used to reconstruct new samples. First, images in the gallery are used to train a generalised deep autoencoder. Second, each person's single sample is used to fine-tune a class-specific deep autoencoder (CDA). Third, the corresponding CDA is used to reconstruct new samples. Finally, these reconstructing new samples are used to do the classification task. Gu et al. [25] proposed local robust sparse representation (LRSR) method. It combined a local sparse representation model and a patch-based generic variation dictionary learning model to predict the possible facial intraclass variation of the query images. Ding et al. [26] partitioned the aligned face image into several nonoverlapping patches to form the training set, then utilized a kernel principal component analysis network to obtain filters and feature banks, and at last, used weighted voting method to occur in the identification of the unlabeled probe. Based on a robust representation and probabilistic graph model, Ji et al. [27] proposed an algorithm to address this problem. They used label propagation to construct probabilistic labels for the samples in the generic training set corresponding to those in the gallery set. At the classification stage, a reconstruction-based classifier is used. Inspired by discriminant manifold learning and binary encoding, Zhang et al. [28] constructed local histogram-based facial image descriptors. They partitioned every image into several nonoverlapping patches, found a matrix to project these patches on to an optimal subspace to maximize manifold margins of different people, reshaped each column of the matrix to an image filter to process facial images, and binarized the responses corresponding to these filters according to thresholding. In classification, they computed region-wise histograms of pixels' binary codes and concatenated them to form the representation of tested image. Dong et al. [29] proposed *k* nearest neighbor virtual image set-based multimanifold discriminant learning method. They put forward a virtual sample generating algorithm to enrich intraclass variation information for training samples inspired by the fact that similar faces have similar intraclass variations. Otherwise, they come up with image set-based multimanifold discriminant learning algorithm to use the intraclass variation information.

However, most of these methods are traditional methods, and there are few DL methods which are very active in CV recently and have a good performance in CV task. Gao et al. [12] proposed a DL method to solve the SSPP FR problem via learning deep supervised autoencoders. Firstly, a supervised autoencoder enforced facial variations to be mapped with canonical face of the same person and enforced the features of the same person to be similar. Then, such supervised autoencoders were stacked to obtain deep architecture. Finally, the supervised autoencoder with deep architecture was used to extract features. Recently, there is no DCNN method to process this task, but due to its good performance in CV, it will become a promising method.

## 3. Expanding Sample Method

**Figure 2:**
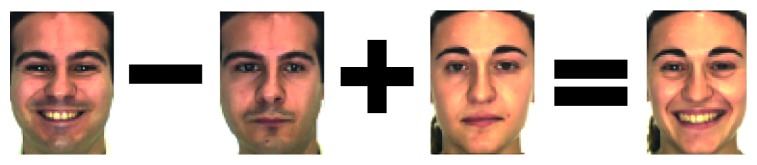
The basic principle diagram of the expanding sample method.

In order to overcome the lack of the training sample in SSPP FR, we propose an expanding sample method. It firstly learns an intraclass variation set, and then the intraclass variation set is added to single sample to expand sample. Its principle diagram is illustrated in Figure 2.

The details of generating intraclass variation set are as follows.

First, generate intraclass variation images according to images of an extrafrontal face dataset. Suppose that there are *m* subjects in an extrafrontal face dataset, each subject has (*n* − 1) variation images and one neutral image, so we can use *X* to express the dataset; let *X*_*ij*_ represent the *i*th person's *j*th variation image, where *i* ∈ [1, *m*], *j* ∈ [1, *n*], and let *j*=1 represent the neutral face. We use variation image of the database (*X*_*ij*_, *j* ≠ 1) minus its corresponding neutral image (*X*_*i*1_); thus, we get variance of the variation image relative to its neutral image, as follows:

(1) $$\begin{array}{c} \varepsilon_{ij}=X_{ij}-X_{i1},\;j\neq 1, \end{array}$$

which represents the *i*th subject's *j*th intraclass variation image relating to its neutral image.

Then, find the average intraclass variation image that has the same variation in these intraclass variation images to decrease the error of intraclass variation image, as follows:

(2) $$\begin{array}{c} \bar{\varepsilon_{j}}=\frac{1}{m}\overset{m}{\underset{i=1}{\sum }}\varepsilon_{ij}. \end{array}$$

Finally, construct an intraclass variation set according to these learned average intraclass variation images in the forward step. It is shown as follows:

(3) $$\begin{array}{c} \bar{\varepsilon }=\left(\bar{\varepsilon_{2}},\bar{\varepsilon_{3}},...,\bar{\varepsilon_{n}}\right). \end{array}$$

The specific steps of generating intraclass variation set are summarized in Table 1.

**Figure 3:**
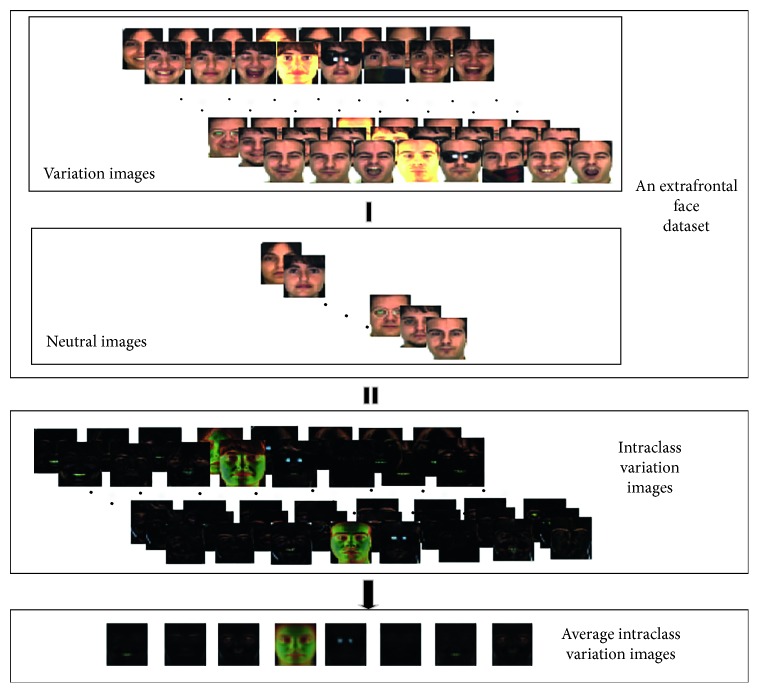
The framework of generating intraclass variation set.

The framework of generating intraclass variation set is illustrated in Figure 3.

Later, with the help of C++ and MATLAB, the face image is detected and cropped from the new input face image, and then the face image is resized to the same size with the intraclass variation set. At last, the intraclass variation set is added to the aligned face image for expanding image as follows:

(4) $$\begin{array}{c} D_{\text{e}k}=\bar{\varepsilon }+X_{k1}, \end{array}$$

where *X*_*k*1_ represents the neutral face image of the person *k* and *D*_e*k*_ represents the expanding samples of the person *k*.

According to the method, single sample is expanded to many samples.

**Figure 4:**
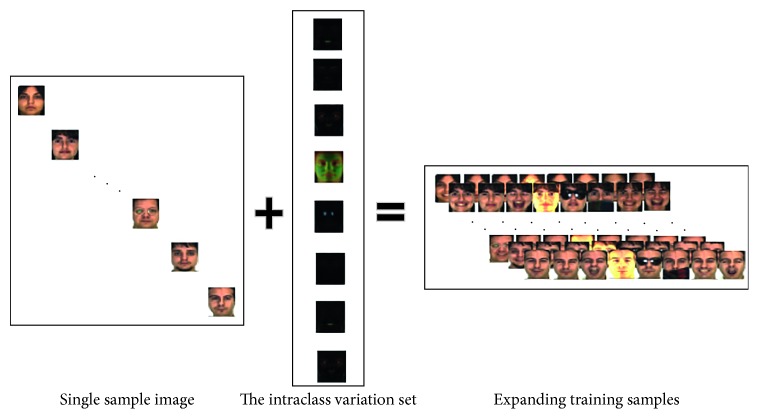
The framework of expanding sample.

The framework of expanding sample is shown in Figure 4.

## 4. Deep Learning Method

As DCNN needs a large amount of samples to be trained, it is difficult to be used in SSPP FR. In order to solve this problem, firstly, we use transfer learning to introduce a well-trained DCNN. Then, we select some expanding samples to fine-tune the learned DCNN. Finally, we use the fine-tuned DCNN to implement experiment.

### 4.1. Transfer Learning

Transfer learning uses knowledge learned from one specific scene to help another application scenario. In other words, it uses auxiliary data to learn a model or mapping and then uses the model or mapping to do a new task.

Since there is one training sample in SSPP FR, DCNN which needs abundant training data is difficult to be used. Therefore, we use transfer learning to introduce a well-trained DCNN model. Here, we have the aid of a lightened CNN [30] which can learn a compact embedding for face recognition to do the research.

Different from other DCNN models, the lightened CNN introduces a new activated function named Max-Feature-Map which introduces maxout in the fully connected layer to the convolution layer. Given an input convolution layer *C* ∈ *R*^*h*×*w*×2*n*^, the Max-Feature-Map activation function can be written as follows:

(5) $$\begin{array}{c} f_{ij}^{k}=\underset{1\leq n}{\max}\left(C_{ij}^{k},C_{ij}^{k+n}\right), \end{array}$$

where the channel of the input convolution layer is 2*n*, *i* ∈ [1, *h*], *j* ∈ [1, *w*].

**Figure 5:**
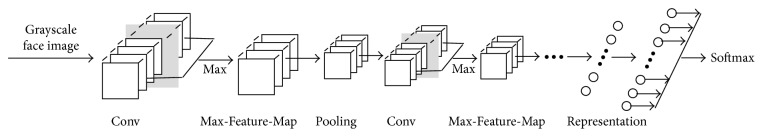
The architecture of the lightened CNN.

The architecture of the lightened CNN is illustrated in Figure 5.

### 4.2. Fine-Tuning

The lightened CNN is trained by CASIA-WebFace database. The CASIA-WebFace database contains 10,575 persons and has a total of 493,456 face images. Before it is used to train the lightened CNN, it is firstly preprocessed. The preprocessing includes the images that are converted to grayscale images and normalized to 144 × 144. After it is preprocessed, it is used to train the lightened CNN. Later, a well-trained model is obtained. We use the well-trained model to do the fine-tuning task. Some expanding samples are selected and put into the well-trained model to do fine-tuning. And the fine-tuned model is used to implement experiment.

## 5. Experiments

We test the performance of TDL on AR face database [31], Extend Yale B face database [32], FERET database [33], and LFW face database [34], respectively. We also compare TDL with the following methods:

- Direct method: SRC [35], CRC [36], PCA [37], (PC)2A [38], E (PC)2A [39], 2DPCA [40], (2D)2PCA [41], SOM [42], LPP [43], and UP [44];
- Generic learning method: AGL [45], ESRC [15], SVDL [18], and LGR [6];
- Patch-based method: DMMA [16], PNN [46], PCRC [47], TLC [5], Block PCA [48], Block LDA [49], and Fast DMMA [20];
- Expanding sample method: SVD-LDA [10];
- DL method: SSAE [12].

Since TDL is regarded the proposed method, the expanding sample method is proposed for TDL, so when these methods are used to be compared, these are not using the generated training images. But the expanding sample method has been demonstrated that it has a good performance compared with the direct method [50].

### 5.1. Similarity

Here, we use AR face database to produce intraclass variation set. To describe briefly, the expanding images are numbered as 1, 2, 3,…, 26 based on their types of variation. Their meanings are described as follows: 1: neutral expression, 2: smile, 3: anger, 4: scream, 5: left light on, 6: right light on, 7: all side light on, 8: wearing sunglasses, 9: wearing sunglasses and left light on, 10: wearing sunglasses and right light on, 11: wearing scarf, 12: wearing scarf and left light on, 13: wearing scarf and right light on, and 14 to 26: same conditions as 1 to 13 but not in the same period. We divide these images into two sessions, session 1 and session 2. Session 1 includes 1 to 13, and session 2 includes 14 to 26.

In order to evaluate the similarities between expanding samples and actual images, an algorithm is proposed.

The details of measuring similarities between expanding samples and actual images are as follows.

First, calculate the Euclidean distances between expanding samples and actual images *E*_d_. Suppose that there are *m* persons and *n* variations, we label expanding samples as *D*_e_ and label actual samples as *D*_a_. We use every pixel of the *i*th person's image with the *j*th variation in expanding samples *D*_*eij*_ minus the corresponding pixel of the *i*th person's image with the *j*th variation in actual images *D*_*aij*_. So we get the Euclidean distance of the *i*th person with the *j*th variation image between expanding sample and actual image *E*_*dij*_, as follows:

(6) $$\begin{array}{c} E_{dij}=D_{eij}-D_{aij}, \end{array}$$

where *i* ∈ [1, *m*], *j* ∈ [1, *n*].

Second, calculate average Euclidean distance of the *j*th variation $\bar{E_{dj}}$ which is used as the threshold of the *j*th intraclass variation, as follows:

(7) $$\begin{array}{c} \bar{E_{dj}}=\frac{1}{m}\overset{m}{\underset{i=1}{\sum }}E_{dij}. \end{array}$$

Third, count the number of similar images. Let *N*_*j*_ represent the similar number of the *j*th variation image. When the Euclidean distance *E*_*dij*_ is bigger than the threshold of intraclass variation $\bar{E_{dj}}$, it is regarded that the expanding sample is not similar to the actual image. Otherwise, it is similar as follows:

(8) $$\begin{array}{c} E_{dij}\leq \bar{E_{dj}},\;\text{similar},\\ E_{dij}>\bar{E_{dj}},\;\text{not similar}. \end{array}$$

Finally, calculate the similarity of the *j*th variation between expanding samples and actual samples *η*_*j*_, as follows:

(9) $$\begin{array}{c} \eta_{j}=\frac{N_{j}}{m}\times 100\%. \end{array}$$

Its specific steps are shown in Table 2.

The thresholds of intraclass variation and the similarities are shown in Tables 3 and 4, respectively.

### 5.2. Intraclass Variation Set

In Table 4, we can see several similarities are very low, which may be detrimental to the experimental results, so it is necessary to select the best intraclass variation set.

We label these expanding samples as Part I, Part II, Part III, and Part IV according to the similarity that is no less than 90%, 95%, 99%, and 100%, respectively. Then, we can know that Part I includes 1, 2, 3, 4, 5, 6, 7, 9, 10, 14, 15, 16, 17, 18, 19, 20, 22, and 23. Part II includes 1, 2, 3, 4, 5, 6, 7, 9, 10, 14, 15, 16, 18, 19, and 20. Part III includes 1, 2, 3, 4, 5, 6, and 7. Part IV includes 1, 2, 3, 5, 6, and 7. We also label Part V which includes all expanding samples and label Part VI which includes SSPP. So it can be known that the number of samples in Part I, Part II, Part III, Part IV, Part V, and Part VI is 1800, 1500, 700, 600, 2600, and 100, respectively.

**Figure 6:**
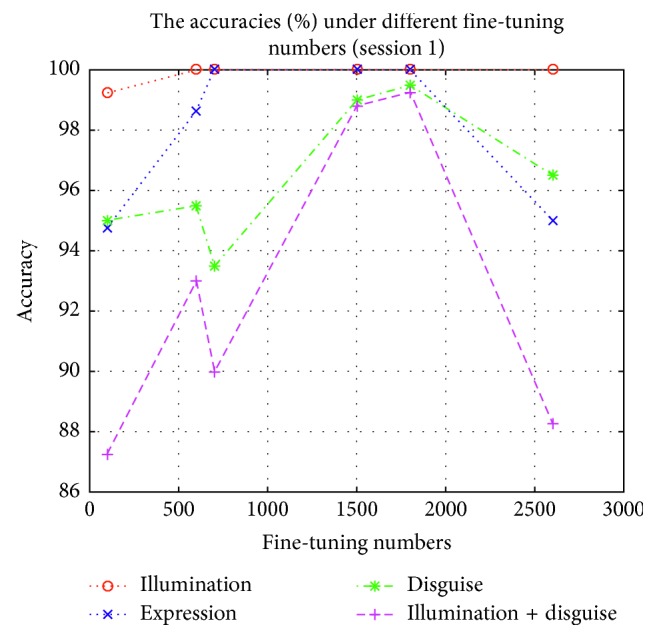
The accuracies in session 1 by using different parts to fine-tune the lightened CNN.

**Figure 7:**
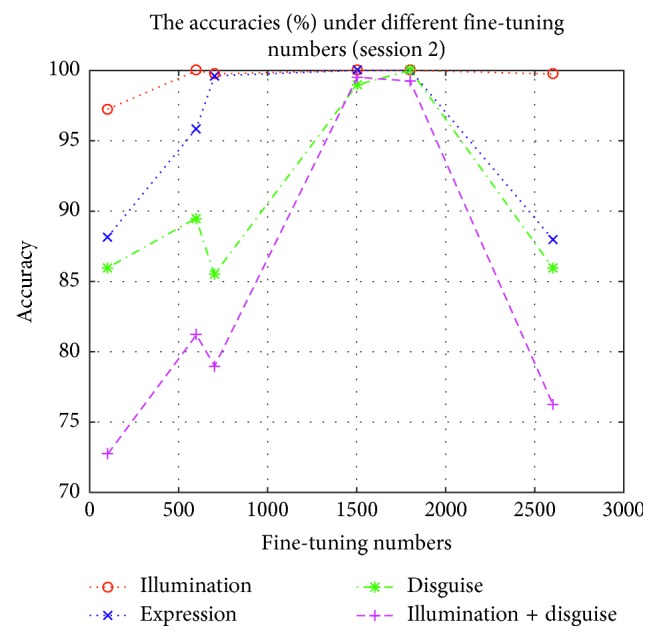
The accuracies in session 2 by using different parts to fine-tune the lightened CNN.

**Figure 8:**
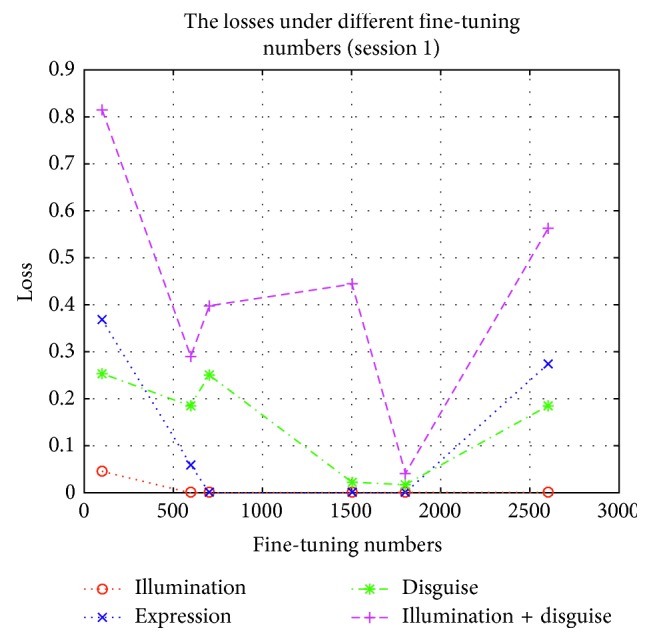
The losses in session 1 by using different parts to fine-tune the lightened CNN.

**Figure 9:**
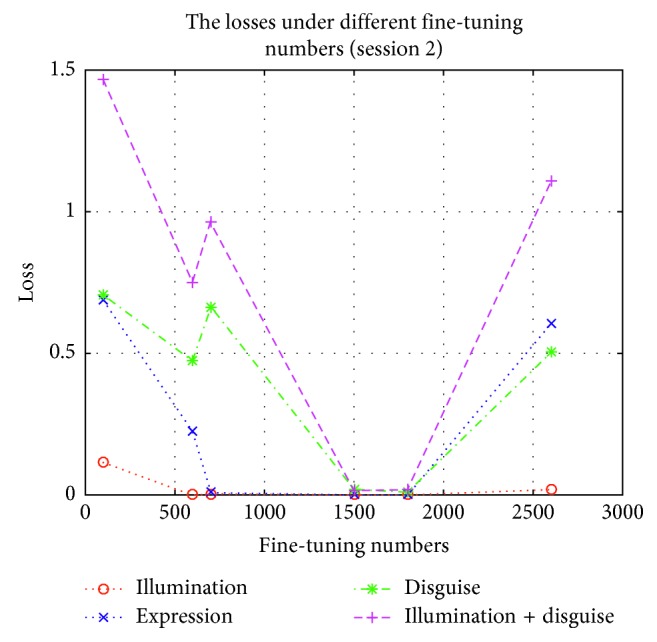
The losses in session 2 by using different parts to fine-tune the lightened CNN.

In order to test the influence of these expanding samples, we test the accuracies and losses in session 1 and session 2 by using Part I, Part II, Part III, Part IV, Part V, and Part VI to fine-tune the lightened CNN model, respectively. These fine-tuned models are used to implement experiment on AR face database, respectively. The accuracies and losses are shown in Figures 6–9, respectively.

According to Figures 6–9, we can find that the accuracies in Figure 6 are the highest when the fine-tuning number is 1800, so does in Figure 7. We also find the errors in Figure 8 are the lowest when the fine-tuning number is 1800, so does in Figure 9. All in all, Part I is selected to implement experiment. Correspondingly, these models which are used to produce Part I is selected as the final version of intraclass variation set.

So we can know that these models are these variation types, as follows: 1: neutral expression, 2: smile, 3: anger, 4: scream, 5: left light on, 6: right light on, 7: all side light on, 9: wearing sunglasses and left light on, 10: wearing sunglasses and right light on, 14: neutral expression, 15: smile, 16: anger, 17: scream, 18: left light on, 19: right light on, 20: all side light on, 22: wearing sunglasses and left light on, and 23: wearing sunglasses and right light on.

### 5.3. AR Face Database

AR face database consists of 126 persons (70 men and 56 women) with more than 4,000 color face images. These images were taken in two-week interval and were divided into two sessions which were session 1 and session 2. In the experiment, a face subdatabase including 50 men and 50 women is selected.

We use Part I to fine-tune the lightened CNN. Then the fine-tuned model is used to perform experiment. The accuracies of different methods in session 1 and session 2 are shown in Tables 5 and 6, respectively.

We can see from Table 5 that the direct method has a poorest performance among these methods, and patch-based method is better than generic learning method. The patch-based method TLC outperforms the generic learning method LGR by 0.4%, 0.6%, and 1.8% under expression, disguise, and illumination with disguise conditions, respectively. But under the same conditions, TDL outperforms TLC by 1.7%, 0.1%, and 1.2%, respectively. Besides, we find that the accuracies under expression and illumination conditions achieve 100%.

In Table 6, we can find that the patch-based method TLC is very competitive, and it outperforms the generic learning method LGR by 1.7%, 2.1%, 2.5%, and 3.1% under different conditions, but the proposed TDL outperforms TLC by 0.8%, 12.9%, 3.7%, and 7.4%, respectively. Especially, the accuracies obtained by using TDL achieve 100% under illumination, expression, and disguise conditions.

The accuracies in Table 5 and Table 6 are very high. On the one hand, it is because the images in AR face database were taken under strictly controlled conditions. On the other hand, the intraclass variation set has the same variations as the images of AR face database.

### 5.4. Extend Yale B Face Database

Extend Yale B face database contains 38 subjects, and each subject has 64 images under different pose and illumination conditions. Different from other experiments that using one part of the database as testing samples and another as generic samples and training samples, in the experiment, the intraclass variation set is added to the neutral and normal illumination image of each subject to obtain adequate training samples, and the rest of the database is used as testing samples. These expanding samples are used to fine-tune the well-trained DCNN model, and then the fine-tuned model is used to perform experiment. The accuracies obtained by using different methods are shown in Table 7.

We can find that the direct method still has the lowest recognition rate and DL method SSAE is better than direct method; however, the generic learning methods SVDL and LGR outperform SSAE by 2.8% and 4.4%, respectively. But TDL outperforms SVDL and LGR by 3.3% and 1.7%, respectively. We also find that the accuracy on Extend Yale B face database is lower than that on AR face database. For one thing, these expanding samples have no same variation as testing samples. For another, Extend Yale B face database has a greater degree of change corresponding to its neutral images compared with AR face database.

### 5.5. FERET Face Database

FERET face database contains 200 subjects with 1400 images under different pose, expression, and illumination conditions. The neutral and normal image of each person is used as single sample to expand sample by adding the intraclass variation set to it. The rest is used as testing samples. These expanding samples are used to fine-tune the DCNN model. Then, the fine-tuned DCNN model is applied to implement experiment.  Table 8 lists the accuracies of different methods.

We can see from Table 8 that the direct method consistently performs worst than other methods. Expanding sample method also exhibits worse results. The expanding sample method SVD-LDA outperforms the direct method PCA by 1.5%; however, the best direct method SOM outperforms SVD-LDA by 5.5%, but the patch-based method DMMA outperforms SOM by 2%. The proposed method TDL achieves the best performance and outperforms the second DMMA by 0.9%.

### 5.6. LFW Database

The LFW database contains 1680 subjects with more than 13000 images which were collected from Web and had many unconstrained conditions. Followed by [6], LFW-a is used to implement experiment. We select 50 persons from LFW-a who have more than 10 images to do experiment. These images are preprocessed before being used. First, the face images are cropped. Second, the cropped face images are resized to 144 × 144. Third, the intraclass variation set is added to one image of each person to get more training samples. Finally, these expanding samples are used to fine-tune the DCNN model, and then the remaining images of the database are tested on the fine-tuned model.  Table 9 presents the accuracies obtained by different methods.

We can find that all the accuracies are very low and none of them overtakes 31%; however, the proposed method TDL achieves the best which is 74% and outperforms the second LGR by 43.6% more than 2 times. Particularly, the LFW database is taken under unconstrained conditions. The experimental result proves that although the intraclass variation set is obtained by constrained images, it also can be used in unconstrained conditions.

From Tables 7–9, we can find that TDL has the best performance compared with other method, although the intraclass variation set is obtained by another database. On the one hand, it demonstrates that the intraclass variation set has a wide range of practicability. On the other hand, it shows that TDL has a better generic ability.

From Tables 5–9, we find that the direct method is the poorest method, expanding sample method is the second poorest method, generic learning method is more better than expanding sample method, patch-based method is the best method among these methods, and the DL method SSAE performs worse than generic learning method, but the proposed method TDL is better than patch-based method. It says that TDL not only outperforms expanding sample method but also has a better performance compared with direct method, generic method, patch-based method, and another DL method. Otherwise, we also find that recognition rates on AR face database are very high which is because the intraclass variation is learned from the same database, recognition rate on LFW database is the lowest among these database which is because the assumption of the model is to deal with frontal faces, so the final system is only working with frontal faces, when it is tested on LFW database which concludes nonfrontal faces the recognition rate dropped sharply.

## 6. Conclusion and Future Work

In this paper, we propose a scheme combined traditional and DL (TDL) method for single sample per person (SSPP) face recognition (FR). First, a novel expanding sample method is proposed to increase training sample. Second, similarities between expanding samples and actual samples are validated, and then the best intraclass variation set is selected as expanding sample model based on the similarity and performance on these actual samples. Third, the selected intraclass variation set is used to expand training sample, and then the DCNN model is fine-tuned. Finally, experiments are implemented on the fine-tuned DCNN model. Extensive experimental results on several databases including AR face database, Extend Yale B face database, FERET face database, and LFW database demonstrate that TDL achieves the state-of-the-art performance among these methods in SSPP FR. Besides, this paper is a pioneer that uses DCNN in SSPP FR, which makes it possible that DCNN is used in single sample or few samples.

In the future, on the one hand, a research on how to improve its accuracy and practicability will be continued, and on the other hand, a research on how to strictly carry out the alignment between the new image and the reference images will also be continued.

## Figures and Tables

**Table 1:** The algorithm of generating intraclass variation set.

Input: an extrafrontal face dataset *X*
Output: intraclass variation set $\bar{\varepsilon }$
(1) calculate:
** ** *ε*_*ij*_=*X*_*ij*_ − *X*_*i*1_
where *i* ∈ [1, *m*], *j* ∈ [2, *n*].
(2) calculate:
** ** $\bar{\varepsilon_{j}}=\left(1/m\right)\sum_{i=1}^{m}\varepsilon_{ij}$
(3) output intraclass variation set, as follows:
** ** $\bar{\varepsilon }=\left(\bar{\varepsilon_{2}},\bar{\varepsilon_{3}},...\bar{\varepsilon_{n}}\right)$

**Table 2:** The algorithm of measuring similarity between expanding samples and actual images.

Input: expanding samples *D*_e_, actual samples *D*_a_
Output: the similarity of the *j*th variation image between expanding samples and actual samples *η*_*j*_
1. Calculate *E*_*dij*_=*D*_*eij*_ − *D*_*aij*_
2. Calculate $\bar{E_{dj}}=\left(1/m\right)\overset{m}{\underset{i=1}{\sum }}E_{dij}$
3. Initialize *N*_*j*_=0
4. for (*i*=1; *i* ≤ *m*; *i*++)
** **if $\left(E_{dij}\leq \bar{E_{dj}}\right)$
** ** *N*_*j*_=*N*_*j*_+1;
** **else
** ** *N*_*j*_=*N*_*j*_;
** **end
5. Calculate *η*_*j*_=(*N*_*j*_/*m*) × 100%

**Table 3:** The thresholds of intraclass variation.

Number	2	3	4	5	6
Threshold	802.3	814.1	873.0	839.5	804.3
Number	7	8	9	10	11
Threshold	834.0	855.1	914.1	898.5	636.6
Number	12	13	14	15	16
Threshold	780.9	835.8	815.2	848.9	880.1
Number	17	18	19	20	21
Threshold	889.9	864.3	850.0	856.3	895.4
Number	22	23	24	25	26
Threshold	953.5	945.2	614.4	793.9	804.4

**Table 4:** The similarities between expanding database and AR database.

Number	1	2	3	4	5	6
Similarity	100%	100%	100%	99%	100%	100%
Number	7	8	9	10	11	12
Similarity	100%	88%	98%	98%	4%	26%
Number	13	14	15	16	17	18
Similarity	40%	98%	97%	98%	94%	98%
Number	19	20	21	22	23	24
Similarity	98%	97%	80%	94%	93%	1%
Number	25	26	—	—	—	—
Similarity	29%	29%	—	—	—	—

**Table 5:** Accuracy (%) on AR face database (session 1).

Method	Illu	Exp	Dis	Disill
SRC [35]	80.8	85.4	55.6	25.3
CRC [36]	80.5	80.4	58.1	23.8
AGL [45]	93.3	77.9	70.0	53.8
DMMA [16]	92.1	81.4	46.9	30.9
PNN [46]	84.6	86.7	90.0	72.5
PCRC [47]	95.0	86.7	95.6	81.3
ESRC [15]	99.6	85.0	83.1	68.6
SVDL [18]	98.3	86.3	86.3	79.4
LGR [6]	100	97.9	98.8	96.3
TLC [5]	100	98.3	99.4	98.1
TDL	100	100	99.5	99.3

Illu,   illumination; Exp,   expression; Dis,  disguise; Disill, illumination + disguise.

**Table 6:** Accuracy (%) on AR face database (session 2).

Method	Illu	Exp	Dis	Disill
SRC [35]	55.8	68.8	29.4	12.8
CRC [36]	55.8	69.6	35.0	13.5
AGL [45]	70.8	55.8	40.6	30.7
DMMA [16]	77.9	61.7	28.1	21.9
PNN [46]	77.5	73.8	71.9	52.8
PCRC [47]	88.8	71.7	81.8	63.1
ESRC [15]	87.9	70.4	59.4	45.0
SVDL [18]	87.1	74.2	61.3	54.1
LGR [6]	97.5	85.0	93.8	88.8
TLC [5]	99.2	87.1	96.3	91.9
TDL	100	100	100	99.3

**Table 7:** Accuracy on Extend Yale B face database.

Method	Accuracy (%)
SRC [35]	49.2
CRC [36]	51.2
AGL [10]	59.5
DMMA [16]	61.7
PNN [46]	67.5
PCRC [47]	77.8
ESRC [15]	67.9
SSAE [12]	82.2
SVDL [18]	85.0
LGR [6]	86.6
TDL	88.3

**Table 8:** Accuracy on FERET database.

Method	Accuracy (%)
PCA [37]	84.0
(PC)^2^A [38]	84.5
E (PC)^2^A [39]	85.5
2DPCA [40]	84.5
(2D)^2^PCA [41]	85.0
SOM [42]	91.0
LPP [43]	84.0
SVD - LDA [10]	85.5
Block PCA [48]	84.5
Block LDA [49]	86.5
UP [44]	90.0
DMMA [16]	93.0
Fast DMMA [20]	91.0
TDL	93.9

**Table 9:** Accuracy on LFW database.

Method	Accuracy (%)
SRC [35]	20.4
CRC [36]	19.8
AGL [45]	19.2
DMMA [16]	17.8
PNN [46]	17.6
PCRC [47]	24.2
ESRC [15]	27.3
SVDL [18]	28.6
LGR [6]	30.4
TDL	74
